# Oral Anticoagulants in Very Elderly Nonvalvular Atrial Fibrillation Patients With High Bleeding Risks: ANAFIE Registry

**DOI:** 10.1016/j.jacasi.2022.07.008

**Published:** 2022-11-15

**Authors:** Ken Okumura, Takeshi Yamashita, Masaharu Akao, Hirotsugu Atarashi, Takanori Ikeda, Yukihiro Koretsune, Wataru Shimizu, Shinya Suzuki, Hiroyuki Tsutsui, Kazunori Toyoda, Atsushi Hirayama, Masahiro Yasaka, Takenori Yamaguchi, Satoshi Teramukai, Tetsuya Kimura, Yoshiyuki Morishima, Atsushi Takita, Hiroshi Inoue

**Affiliations:** a Division of Cardiology, Saiseikai Kumamoto Hospital, Kumamoto, Japan; b The Cardiovascular Institute, Tokyo, Japan; c Department of Cardiology, National Hospital Organization Kyoto Medical Center, Kyoto, Japan; d AOI Hachioji Hospital, Tokyo, Japan; e Department of Cardiovascular Medicine, Toho University Faculty of Medicine, Tokyo, Japan; f National Hospital Organization Osaka National Hospital, Osaka, Japan; g Department of Cardiovascular Medicine, Graduate School of Medicine, Nippon Medical School, Tokyo, Japan; h Department of Cardiovascular Medicine, Faculty of Medical Sciences, Kyushu University, Fukuoka, Japan; i Department of Cerebrovascular Medicine, National Cerebral and Cardiovascular Center, Osaka, Japan; j Osaka Police Hospital, Osaka, Japan; k Department of Cerebrovascular Medicine and Neurology, Cerebrovascular Center, National Hospital Organization Kyushu Medical Center, Fukuoka, Japan; l Department of Biostatistics, Graduate School of Medical Science, Kyoto Prefectural University of Medicine, Kyoto, Japan; m Primary Medical Science Department, Daiichi Sankyo Co, Ltd, Tokyo, Japan; n Data Intelligence Department, Daiichi Sankyo Co, Ltd, Tokyo, Japan; o Saiseikai Toyama Hospital, Toyama, Japan

**Keywords:** anticoagulants, atrial fibrillation, high risk, outcomes, very elderly, AF, atrial fibrillation, CrCl, creatinine clearance, DOAC, direct-acting oral anticoagulant, NVAF, nonvalvular atrial fibrillation, OAC, oral anticoagulant, SE, systemic embolism, TTR, time in the therapeutic range

## Abstract

**Background:**

Data on the effectiveness and safety of oral anticoagulant (OAC) agents in very elderly nonvalvular atrial fibrillation patients with high bleeding risk are lacking.

**Objectives:**

This study examined 2-year outcomes and effects of OAC agents among these patients using the ANAFIE (All Nippon Atrial Fibrillation in the Elderly) registry (N = 32,275) data.

**Methods:**

Patients were classified into high-risk (age: ≥80 years; CHADS_2_ score: ≥2; and presence of ≥1 bleeding risk factor: creatinine clearance of 15-30 mL/minute, prior bleeding at critical sites, body weight of ≤45 kg, or continuous antiplatelet use) and reference groups.

**Results:**

In the high-risk (n = 7,104) and reference (n = 25,171) group patients, 89.0% and 93.4%, respectively, used OAC agents. Of these, respectively, 30.1% and 24.2% used warfarin, and 58.9% and 69.1% used direct-acting OAC (DOAC) agents. Compared with the reference group, the high-risk group had higher incidences of stroke/systemic embolism, major bleeding, intracranial hemorrhage, gastrointestinal bleeding, cardiovascular events, and all-cause death. In the high-risk group, DOAC agent use vs nonuse of OAC agents was associated with reduced incidences of stroke/systemic embolism (HR: 0.53; 95% CI: 0.36-0.79) and all-cause death (HR: 0.65; 95% CI: 0.52-0.81) but not with major bleeding (HR: 1.09; 95% CI: 0.63-1.89). DOAC agents were superior to warfarin in effectiveness and safety. For high-risk patients, history of major bleeding, severe liver dysfunction, and falls within 1 year were independent risk factors for major bleeding.

**Conclusions:**

High-risk elderly nonvalvular atrial fibrillation patients had higher event incidences. DOAC agents were associated with reduced risk of stroke/systemic embolism and all-cause death vs nonuse of OAC agents or warfarin. (Prospective Observational Study in Late-Stage Elderly Patients With Nonvalvular Atrial Fibrillation [ANAFIE registry]; UMIN000024006)

Worldwide, atrial fibrillation (AF) is the most common sustained arrhythmia affecting adults, with more than 33 million affected individuals,^1^ and is considered a well established and growing global epidemic.^2^ AF is associated with high morbidity and mortality among those affected. Because aging is the most important risk factor for developing AF,^3^ AF-related complications, such as cardiac dysfunction and stroke,^4^ and their sequelae are expected to increase.

Therapeutic and management strategies and stroke prevention are evolving and improving.^5^ Based on the results of large-scale clinical trials, various guidelines recommend administering oral anticoagulant (OAC) agents, especially direct-acting OAC (DOAC) agents, to prevent stroke in patients with AF, including elderly patients.^6^ It is, however, necessary to rigorously evaluate the current status of OAC agent use and outcomes among very elderly patients, who are generally excluded from most clinical trials. Additionally, treating very elderly patients with AF poses a considerable challenge, not only because of the aging process and its consequences but because of comorbidities, associated complications,^3^ and increased risk of bleeding.^7^^,^^8^

The recent ELDERCARE-AF (Edoxaban Low-Dose for Elder Care Atrial Fibrillation Patients) trial showed that once-daily, very-low-dose edoxaban (15 mg) was superior to placebo in reducing stroke/systemic embolism (SE) in AF patients aged ≥80 years who were at high risk of bleeding and possibly ineligible for standard OAC therapy, and nonsignificantly increased major bleeding.^9^ Nevertheless, there is insufficient real-world evidence of the effectiveness and safety of OAC agents in nonvalvular AF (NVAF) patients who meet the ELDERCARE-AF eligibility criteria, that is, very elderly patients with high bleeding risk. The purposes of the present exploratory subanalysis of the ANAFIE (All Nippon AF in the Elderly) registry were to examine the prescription rate of OAC agents and the 2-year rate of events of interest among high-bleeding-risk patients and to describe the effect of OAC agents in this high-bleeding-risk population.^9^

## Methods

### Study design and population

The ANAFIE registry was a large-scale, prospective, observational, real-world data study of more than 30,000 elderly (≥75 years of age) Japanese patients with NVAF, irrespective of OAC agent use, who were followed up for 2 years.^10^ The rationale, detailed study design, and methodology of the ANAFIE registry (UMIN000024006) have already been published.^11^ The study complied with the Declaration of Helsinki, the locally appointed ethics committees approved the research protocol, and participants provided informed consent.

For the present subanalysis, patients enrolled in the ANAFIE registry were classified into 2 groups, the ELDERCARE-AF–like high-risk group, defined as the patients who met the eligibility criteria for the ELDERCARE-AF trial (high-risk group), and the reference group, defined as the patients who did not meet the ELDERCARE-AF trial criteria.^9^ The criteria for inclusion in the high-risk group were age of ≥80 years; a CHADS_2_ score of ≥2; and the presence of ≥1 bleeding risk factor: creatinine clearance (CrCl) of 15-30 mL/min, except for patients with CrCl of <15 mL/min or undergoing dialysis; prior bleeding history at critical sites (ie, major upper or lower gastrointestinal bleeding or intracranial hemorrhage); body weight of ≤45 kg^12^; and continuous single antiplatelet use (eg, aspirin, P2Y_12_ inhibitor, or other).

### Study measures

Events of interest evaluated in this analysis included the incidences of stroke/SE; major bleeding; all bleeding events (major bleeding, clinically relevant bleeding, and minor bleeding); intracranial hemorrhage; gastrointestinal bleeding; cardiovascular events including stroke/SE, myocardial infarction, heart failure, and cardiovascular death; all-cause death; and net clinical outcome (a composite of stroke/SE, major bleeding, and all-cause death) over the 2-year follow-up period. The events occurring in the high-risk group vs the reference group; the events occurring in the high-risk group according to DOAC agent vs warfarin and DOAC agent vs nonuse of OAC agents; and the independent risk factors for stroke/SE, major bleeding, and intracranial hemorrhage were explored.

DOAC agent doses were defined as previously reported.^13^ Briefly, a standard dose was defined as the dose according to the label specified for patients not meeting the dose-reduction criteria; a reduced dose was the dose according to the label specified for patients who met the dose-reduction criteria; an overdose was the standard dose prescribed to patients who met the reduced-dose criteria; an underdose was the reduced dose prescribed to patients who did not meet the dose-reduction criteria; and an off-label underdose was any dose lower than that specified by the label.

### Statistical analysis

The details of the statistical analysis and sample size calculations of the ANAFIE registry have been reported previously.^11^^,^^14^ Summary statistics were obtained for continuous variables, and a 2-sample Student’s *t*-test was used to calculate *P* values. Categorical variables were summarized using numbers and percentages, and *P* values were calculated using the chi-square test.

The Kaplan-Meier method was used to estimate the cumulative incidence rates of the events of interest at 2 years in the high-risk and reference groups. The log-rank test was used for between-group comparison.

For the comparison of incidence rates in the high-risk vs reference group, the Cox proportional hazards model was used, and HRs and 95% CIs were calculated. In this analysis, anticoagulant therapy was entered in the statistical model. To compare incidence rates between OAC agent use vs nonuse of OAC agents and to identify independent risk factors for stroke/SE, major bleeding, and intracranial hemorrhage, the variables possibly associated with the selection of anticoagulant therapy or incidence of outcomes were entered in the statistical model. All statistical analyses were performed using SAS version 9.4 or higher (SAS Institute).

## Results

### Patients

**Figure 1:**
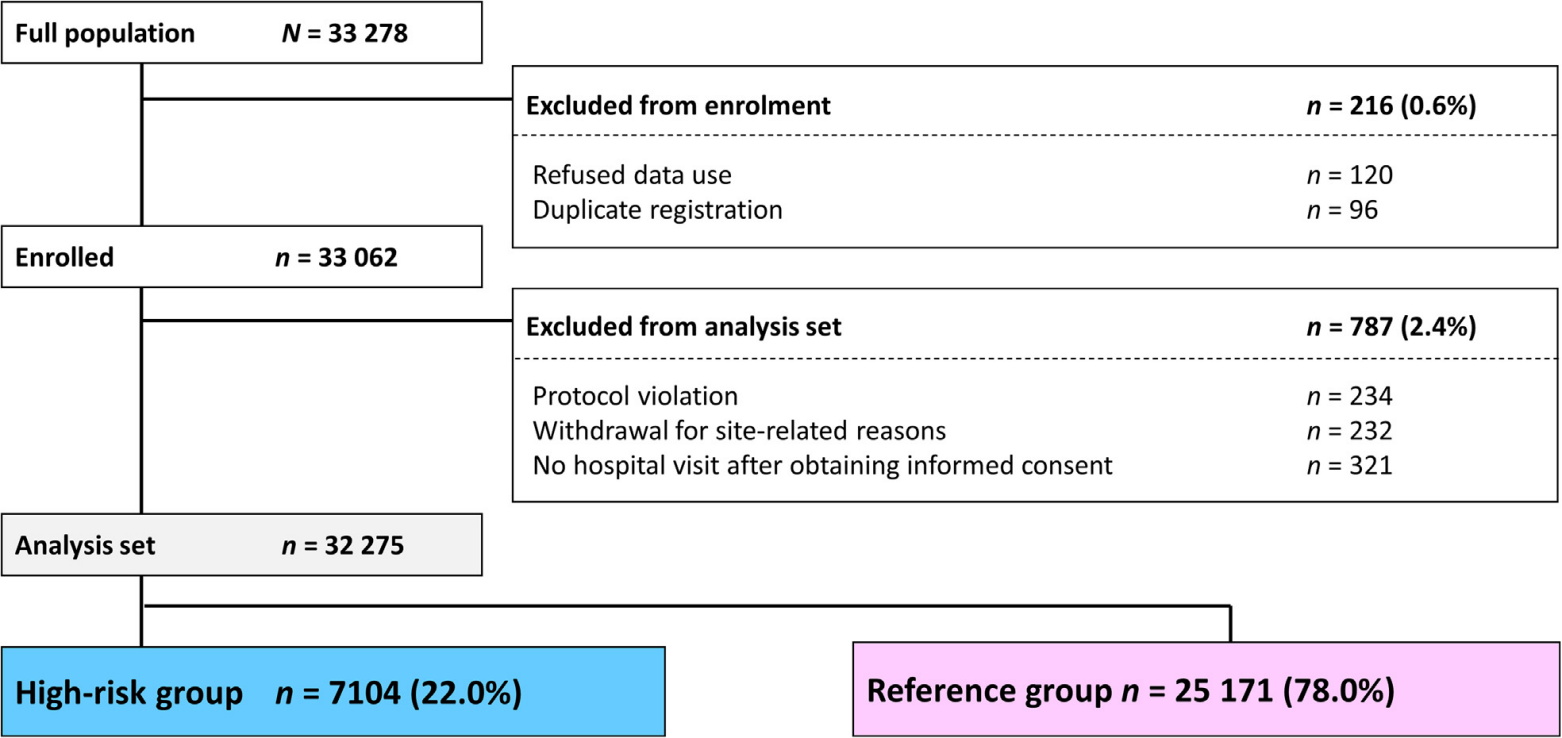
Patient Disposition A total of 32,275 patients were enrolled in the ANAFIE registry, of whom 7,104 (22.0%) met the eligibility criteria for the ELDERCARE-AF trial (high-risk group). These criteria were age of ≥80 years; a CHADS_2_ score of ≥2; and the presence of ≥1 bleeding risk factor: creatinine clearance of 15-30 mL/min; patients with CrCl of <15 mL/minute or undergoing dialysis; prior bleeding history at critical sites (ie, major upper or lower gastrointestinal bleeding or intracranial hemorrhage); body weight of ≤45 kg; and continuous single antiplatelet use (eg, aspirin, P2Y_12_ inhibitor, or other) did not meet the ELDERCARE-AF trial inclusion criteria. The reference group was composed of the 25,171 patients (78.0%) who did not meet the ELDERCARE-AF criteria. ANAFIE = All Nippon Atrial Fibrillation in the Elderly; ELDERCARE-AF = Edoxaban Low-Dose for Elder Care Atrial Fibrillation Patients.

Figure 1 shows the patient disposition. Of the patients enrolled in the ANAFIE registry (N = 32,275), 22.0% (n = 7,104) were included in the high-risk group. The remaining 78.0% (n = 25,171) of patients made up the reference group.

Table 1 summarizes the main characteristics of patients in the high-risk and reference groups. Compared with the reference group, the high-risk group patients were older and had a higher prevalence of female sex, lower body weight, and lower CrCl. The prevalence of OAC agent use was slightly lower in the high-risk group (89.0%) than in the reference group (93.4%), and the rate of warfarin use was higher in the high-risk group (30.1%) than in the reference group (24.2%), whereas the rate of DOAC use was lower in the high-risk group (58.9%) than in the reference group (69.1%). In the high-risk group, 4.4% and 71.5% of patients received DOAC agents at standard doses and on-label reduced doses, respectively; in the reference group, 20.9% and 37.7% of patients were receiving DOAC agents at standard and on-label reduced doses, respectively. The other patients received underdoses (ie, reduced doses not meeting dose-reduction criteria; 7.3% in the high-risk group and 19.1% in the reference group), overdoses (ie, standard doses meeting the dose-reduction criteria; 2.9% in the high-risk group and 3.3% in the reference group), off-label (ie, nonapproved) low doses (5.9% in the high-risk group and 3.2% in the reference group), or unknown doses (8.1% in the high-risk group and 15.8% in the reference group). In patients receiving warfarin, the mean time in the therapeutic range (TTR) was lower in the high-risk group (71.0%) than in the reference group (77.1%); however, almost half of the patients evaluated had a TTR of ≥80% in both groups.

**Table 1:** Patient Characteristics

	ANAFIE Registry (N = 32,275)		
	High-Risk Group (n = 7,104)	Reference Group (n = 25,171)	*P* Value
Men	3,109 (43.8)	15,373 (61.1)	<0.001
Age, y	85.3 ± 4.0	80.4 ± 4.4	<0.001
Body weight, kg	51.2 ± 11.1	59.8 ± 10.4	<0.001
Systolic blood pressure, mm Hg	126.2 ± 18.1	127.7 ± 16.7	<0.001
CrCl, mL/min	35.4 ± 13.9	52.4 ± 17.5	<0.001
CHADS_2_ score	3.2 ± 1.1	2.8 ± 1.2	<0. 001
HAS-BLED score	2.2 ± 0.9	1.8 ± 0.8	<0.001
History of major bleeding	740 (10.4)	699 (2.8)	<0.001
AF type			<0.001
Paroxysmal	2,844 (40.0)	10,742 (42.7)	
Persistent	1,162 (16.4)	4,174 (16.6)	
Long-standing persistent/permanent	3,098 (43.6)	10,255 (40.7)	
Oral anticoagulant agents	6,324 (89.0)	23,506 (93.4)	<0.001
Warfarin	2,139 (30.1)	6,094 (24.2)	<0.001
TTR, %	71.0 ± 31.3	77.1 ± 29.1	<0.001
<40	338 (15.8)	690 (11.3)	
40 to <60	231 (10.8)	490 (8.0)	
60 to <80	329 (15.4)	822 (13.5)	
≥80	935 (43.7)	3,046 (50.0)	
Unknown	306 (14.3)	1,046 (17.2)	
DOAC agents	4,184 (58.9)	17,401 (69.1)	<0.001
Approved doses	3,599 (86.0)	14,103 (81.0)	
Standard doses	183 (4.4)	3,643 (20.9)	
Reduced doses	2,990 (71.5)	6,558 (37.7)	
Overdoses^a^	122 (2.9)	576 (3.3)	
Underdoses^b^	304 (7.3)	3,326 (19.1)	
Off-label low doses	245 (5.9)	550 (3.2)	
Unknown	340 (8.1)	2,748 (15.8)	
Antiplatelet agents	3,024 (42.6)	2,680 (10.6)	<0.001
History of nonpharmacologic therapy for AF	1,072 (15.1)	4,605 (18.3)	<0.001
Catheter ablation	324 (4.6)	2,646 (10.5)	<0.001
Electrical defibrillation	130 (1.8)	585 (2.3)	0.013
Comorbidities	7,067 (99.5)	24,333 (96.7)	<0.001
Hypertension	5,841 (82.2)	18,471 (73.4)	<0.001
Diabetes mellitus	2,037 (28.7)	6,696 (26.6)	<0.001
Chronic kidney disease	2,236 (31.5)	4,469 (17.8)	<0.001
Myocardial infarction	683 (9.6)	1,168 (4.6)	<0.001
Heart failure	3,689 (51.9)	8,427 (33.5)	<0.001
History of cerebrovascular disease	2,130 (30.0)	5,173 (20.6)	<0.001
Gastrointestinal diseases	2,406 (33.9)	7,061 (28.1)	<0.001
Active cancer	737 (10.4)	2,832 (11.3)	0.038
Dementia	948 (13.3)	1,564 (6.2)	<0.001
Fall within 1 year	760 (10.7)	1,587 (6.3)	<0.001

Values are n (%) or mean ± SD.

AF = atrial fibrillation; ANAFIE = All Nippon Atrial Fibrillation in the Elderly; CrCl = creatinine clearance; DOAC = direct-acting oral anticoagulant; TTR = time in the therapeutic range.

a Overdose was defined as the standard dose prescribed to patients who met the reduced-dose criteria.

b Underdose was defined as the reduced dose prescribed to patients who did not meet the reduced-dose criteria.

### Comparison of the incidences of major outcomes by group

**Figure 2:**
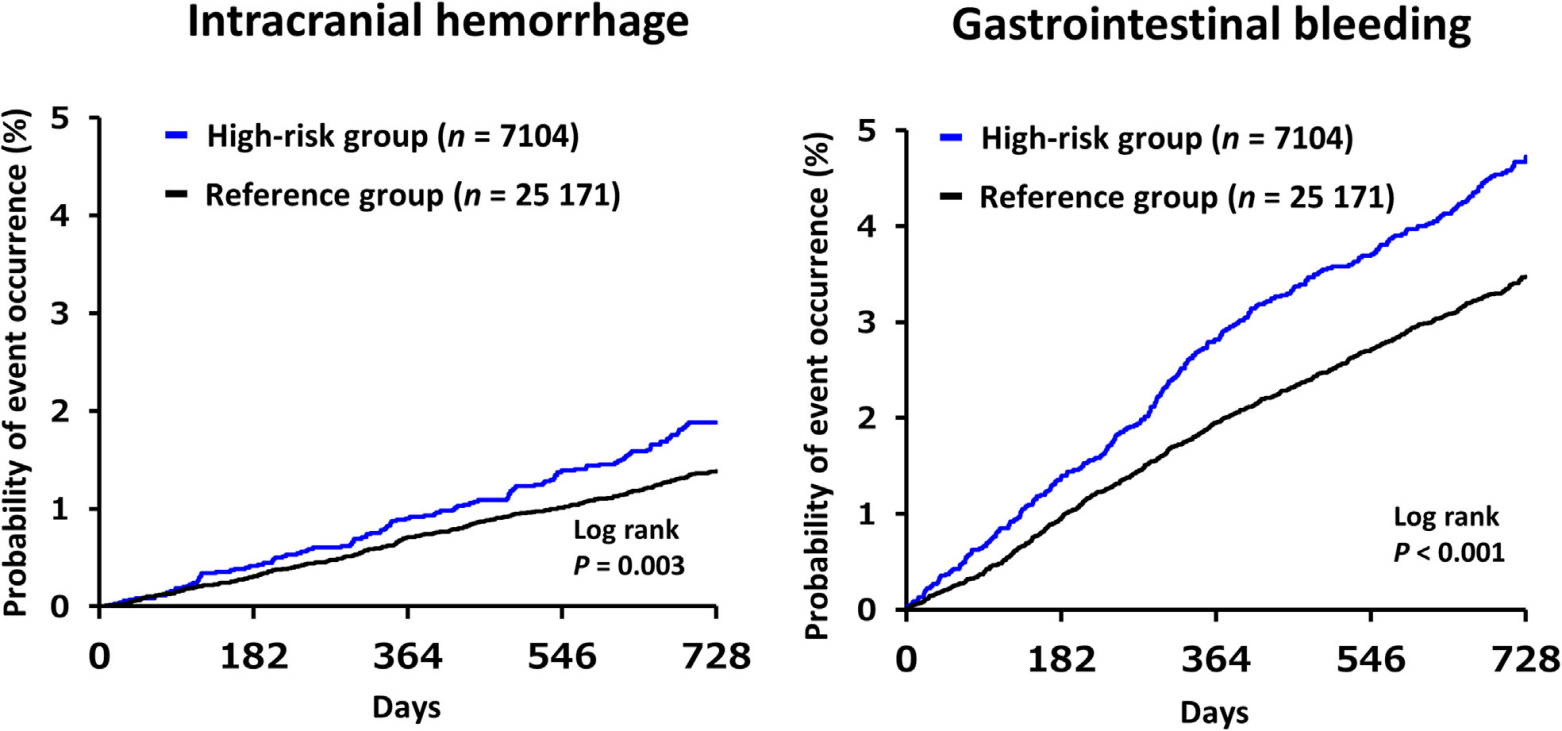
Kaplan-Meier Curves for Intracranial Hemorrhage and Gastrointestinal Bleeding Cumulative incidence rates are shown for intracranial hemorrhage and gastrointestinal bleeding. The high-risk group had higher cumulative incidences of gastrointestinal bleeding (*P* < 0.001) and intracranial hemorrhage (*P* = 0.003) compared with the reference group.

The 2-year incidence rates in the high-risk and reference groups are shown in Table 2. Stroke/SE occurred in 3.8% and 2.8%, respectively; major bleeding in 2.8% and 1.8%, respectively; intracranial hemorrhage in 1.7% and 1.3%, respectively; gastrointestinal bleeding in 4.4% and 3.3%, respectively; cardiovascular events in 17.5% and 8.7%, respectively; and all-cause death in 12.5% and 5.4%, respectively. The cumulative incidence rates of the main outcomes are presented in the Central Illustration and Figure 2. Both univariate and multivariate analyses revealed that compared with the reference group, the high-risk group had higher cumulative incidences of all events (*P* < 0.001 for all events, except for intracranial hemorrhage [*P* = 0.007]).

**Table 2:** Comparison of the Incidences of Major Outcomes by Group^a^

Outcome	Group	Events (%)	Univariate Analysis		Multivariate Analysis^b^	
			HR (95% CI)	*P* Value	HR (95% CI)	*P* Value
Stroke/systemic embolism	High risk	270 (3.8)	1.43 (1.24-1.64)	<0.001	1.39 (1.21-1.60)	<0.001
	Reference	700 (2.8)	—	—	—	—
Stroke	High risk	260 (3.7)	1.41 (1.22-1.62)	<0.001	1.37 (1.18-1.58)	<0.001
	Reference	685 (2.7)	—	—	—	—
Ischemic stroke	High risk	206 (2.9)	1.42 (1.21-1.67)	<0.001	1.37 (1.16-1.61)	<0.001
	Reference	537 (2.1)	—	—	—	—
Hemorrhagic stroke	High risk	51 (0.7)	1.25 (0.91-1.72)	0.162	1.26 (0.91-1.73)	0.161
	Reference	150 (0.6)	—	—	—	—
Systemic embolism	High risk	13 (0.2)	3.16 (1.51-6.65)	0.002	2.96 (1.40-6.25)	0.004
	Reference	15 (0.1)	—	—	—	—
Major bleeding	High risk	198 (2.8)	1.64 (1.39-1.94)	<0.001	1.61 (1.36-1.90)	<0.001
	Reference	447 (1.8)	—	—	—	—
Clinically relevant nonmajor bleeding	High risk	181 (2.6)	1.35 (1.14-1.60)	<0.001	1.36 (1.15-1.62)	<0.001
	Reference	494 (2.0)	—	—	—	—
Minor bleeding	High risk	344 (4.8)	1.23 (1.09-1.39)	0.001	1.25 (1.10-1.41)	<0.001
	Reference	1,036 (4.1)	—	—	—	—
All bleeding events^c^	High risk	675 (9.5)	1.33 (1.22-1.46)	<0.001	1.34 (1.23-1.47)	<0.001
	Reference	1,880 (7.5)	—	—	—	—
Intracranial hemorrhage	High risk	122 (1.7)	1.36 (1.11-1.68)	0.004	1.33 (1.08-1.64)	0.007
	Reference	331 (1.3)	—	—	—	—
Gastrointestinal bleeding	High risk	309 (4.4)	1.38 (1.21-1.57)	<0.001	1.39 (1.22-1.58)	<0.001
	Reference	829 (3.3)	—	—	—	—
Cardiovascular events	High risk	1,240 (17.5)	2.18 (2.03-2.34)	<0.001	2.13 (1.98-2.28)	<0.001
	Reference	2,177 (8.7)	—	—	—	—
All-cause death	High risk	886 (12.5)	2.42 (2.23-2.64)	<0.001	2.33 (2.14-2.54)	<0.001
	Reference	1,355 (5.4)	—	—	—	—
Cardiovascular death	High risk	303 (4.3)	3.20 (2.75-3.74)	<0.001	3.04 (2.61-3.55)	<0.001
	Reference	351 (1.4)	—	—	—	—
Net clinical outcome	High risk	1,148 (16.2)	2.01 (1.88-2.16)	<0.001	1.95 (1.82-2.10)	<0.001
	Reference	2,124 (8.4)	—	—	—	—

a High-risk group: n = 7,104; reference group: n = 25,171.

b Anticoagulant agent use was included as an adjustment factor in the model.

c All bleeding events (major bleeding, clinically relevant bleeding, and minor bleeding).

**Central Illustration:**
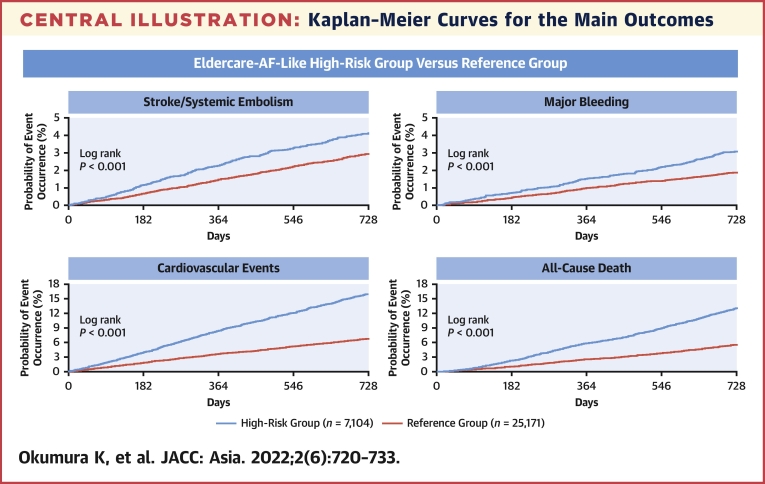
Kaplan–Meier Curves for the Main Outcomes The criteria for inclusion in the ELDERCARE-AF–like high-risk group were age of ≥80 years, a CHADS_2_ score of ≥2, and the presence of ≥1 bleeding risk factor: creatinine clearance of 15-30 mL/min; prior bleeding history at critical sites; body weight of ≤45 kg; and continuous single antiplatelet use. The reference group included all other patients. Cumulative incidence rates are shown for the main outcomes of stroke/systemic embolism, major bleeding, cardiovascular events, and all-cause death. The high-risk group had higher cumulative incidences of all these events compared with the reference group (*P* < 0.001). ELDERCARE-AF **=** Edoxaban Low-Dose for Elder Care Atrial Fibrillation Patients.

Among the high-risk group patients prescribed with DOAC agents, the 2-year incidences of stroke/SE, major bleeding, and all-cause mortality were 3.3%, 2.6%, and 11.6%, respectively, in patients receiving on-label DOAC doses (n = 3,173; standard and reduced doses), whereas the incidences were 2.0%, 2.0%, and 11.5%, respectively, in those receiving inappropriately reduced off-label doses (n = 549; underdoses and off-label low doses) (data not shown).

### Comparison of main outcomes according to anticoagulant agent use in the high-risk group

Table 3 shows the HRs for each event in the Cox proportional hazards model for the high-risk group according to OAC agent use (DOAC agent vs warfarin and vs nonuse of OAC agents) with univariate and multivariate analyses. Stroke/SE, all-cause death, cardiovascular death, and net clinical outcome rates were significantly lower in the DOAC agent–treated group compared with the nonuse-of-OAC-agents group, whereas major bleeding rates did not significantly increase with DOAC agent treatment. The rate for all bleeding events (major bleeding, clinically relevant bleeding, and minor bleeding) was significantly higher in the DOAC agent–treated group than in the nonuse-of-OAC-agents group. Stroke/SE, major bleeding, intracranial hemorrhage, and net clinical outcome rates were significantly lower in the DOAC agent–treated group compared with the warfarin-treated patients, but no significant differences were observed for gastrointestinal bleeding, all-cause death, or cardiovascular death.

**Table 3:** Comparison of Main Outcomes According to Anticoagulant Agent Use in the High-Risk Group

Event	By Type of Anticoagulant Agent	n	Events (%)	Univariate Analysis		Multivariate Analysis^a^	
				HR (95% CI)	*P* Value	HR (95% CI)	*P* Value
Stroke/systemic embolism	Warfarin	2,139	96 (4.5)	Reference		Reference	
	vs						
	DOAC agent	4,184	135 (3.2)	0.71 (0.54-0.92)	0.010	0.73 (0.55-0.95)	0.021
	Nonuse of OAC agent	780	39 (5.0)	Reference		Reference	
	vs						
	DOAC agent	4,184	135 (3.2)	0.62 (0.44-0.89)	0.009	0.53 (0.36-0.78)	0.001
Major bleeding	Warfarin	2,139	76 (3.6)	Reference		Reference	
	vs						
	DOAC agent	4,184	106 (2.5)	0.70 (0.52-0.94)	0.018	0.71 (0.53-0.97)	0.029
	Nonuse of OAC agent	780	16 (2.1)	Reference		Reference	
	vs						
	DOAC agent	4,184	106 (2.5)	1.20 (0.71-2.03)	0.499	1.09 (0.63-1.89)	0.752
All bleeding events^b^	Warfarin	2,139	225 (10.5)	Reference		Reference	
	vs						
	DOAC agent	4,184	405 (9.7)	0.91 (0.77-1.07)	0.233	0.93 (0.79-1.10)	0.421
	Nonuse of OAC agent	780	22 (5.8)	Reference		Reference	
	vs						
	DOAC agent	4,184	405 (9.7)	1.65 (1.22-2.25)	0.001	1.68 (1.22-2.31)	0.001
Intracranial hemorrhage	Warfarin	2,139	51 (2.4)	Reference		Reference	
	vs						
	DOAC agent	4,184	58 (1.4)	0.57 (0.39-0.83)	0.004	0.57 (0.38-0.84)	0.004
	Nonuse of OAC agent	780	13 (1.7)	Reference		Reference	
	vs						
	DOAC agent	4,184	58 (1.4)	0.81 (0.44-1.47)	0.483	0.78 (0.41-1.47)	0.437
Gastrointestinal bleeding	Warfarin	2,139	92 (4.3)	Reference		Reference	
	vs						
	DOAC agent	4,184	192 (4.6)	1.05 (0.82-1.35)	0.680	1.11 (0.86-1.44)	0.406
	Nonuse of OAC agent	780	25 (3.2)	Reference		Reference	
	vs						
	DOAC agent	4,184	192 (4.6)	1.40 (0.92-2.12)	0.114	1.36 (0.88-2.08)	0.165
All-cause death	Warfarin	2,139	300 (14.0)	Reference		Reference	
	vs						
	DOAC agent	4,184	473 (11.3)	0.79 (0.69-0.92)	0.002	0.88 (0.76-1.02)	0.092
	Nonuse of OAC agent	780	113 (14.5)	Reference		Reference	
	vs						
	DOAC agent	4,184	473 (11.3)	0.75 (0.61-0.93)	0.007	0.65 (0.52-0.81)	<0.001
Cardiovascular death	Warfarin	2,139	104 (4.9)	Reference		Reference	
	vs						
	DOAC agent	4,184	161 (3.9)	0.78 (0.61-0.99)	0.044	0.91 (0.70-1.17)	0.445
	Nonuse of OAC agent	780	38 (4.9)	Reference		Reference	
	vs						
	DOAC agent	4,184	161 (3.9)	0.76 (0.54-1.09)	0.133	0.62 (0.43-0.91)	0.014
Net clinical outcome	Warfarin	2,139	394 (18.4)	Reference		Reference	
	vs						
	DOAC agent	4,184	607 (14.5)	0.77 (0.68-0.87)	<0.001	0.83 (0.73-0.95)	0.005
	Nonuse of OAC agent	780	147 (18.9)	Reference		Reference	
	vs						
	DOAC agent	4,184	607 (14.5)	0.74 (0.62-0.88)	0.001	0.62 (0.51-0.75)	<0.001

Abbreviations as in Table 1.

a Sex; age; body mass index; history of bleeding; type of AF; systolic blood pressure; severe liver dysfunction; diabetes mellitus; hyperuricemia; heart failure and/or reduced left ventricular ejection fraction; myocardial infarction; cerebrovascular disease; other thromboembolic disease; active cancer; dementia; fall within 1 year, history of catheter ablation; dyslipidemia; creatinine clearance; gastrointestinal diseases; polypharmacy (5 or more); and use of antiarrhythmic agents, proton pump inhibitors, P-glycoprotein inhibitors, and antiplatelet agents were included as an adjustment factor in the model.

b All bleeding events (major bleeding, clinically relevant bleeding, and minor bleeding).

### Risk factors associated with main outcomes in the high-risk group

Univariate and multivariate analyses of outcomes in the high-risk group according to baseline variables are shown in Table 4. Significant independent risk factors for stroke/SE were type of AF (long-standing AF), systolic blood pressure of ≥140 mm Hg, diabetes (glycated hemoglobin: ≥6.0%), cerebrovascular disease, and CrCl of ≥15 to <30 mL/min. Significant independent risk factors for major bleeding were a history of major bleeding, severe liver dysfunction, and falls within 1 year. Significant independent risk factors of intracranial hemorrhage were severe liver dysfunction, fall within 1 year, and proton pump inhibitor use.

**Table 4:** Univariate and Multivariate Analyses of Outcomes According to Baseline Variables in High-Risk Group

Outcomes and Factors	Variables	n	Events (%)	Univariate Analysis		Multivariate Analysis^a^	
				HR (95% CI)	*P* Value	HR (95% CI)	*P* Value
Stroke/systemic embolism							
Total		7,104	270 (3.8)	－		－	
Sex	Male^b^	3,109	103 (3.3)	－		－	
	Female	3,995	167 (4.2)	1.24 (0.97-1.58)	0.089	1.17 (0.86-1.59)	0.319
Body weight	≤45 kg	2,687	111 (4.1)	1.18 (0.92-1.51)	0.197	1.23 (0.89-1.70)	0.201
	>45 kg^b^	4,088	145 (3.6)	－		－	
History of major bleeding	Yes	681	32 (4.7)	1.27 (0.87-1.83)	0.212	1.26 (0.84-1.90)	0.265
	No^b^	6,423	238 (3.7)	－		－	
Type of AF	Paroxysmal^b^	2,844	79 (2.8)	－		－	
	Persistent	1,162	44 (3.8)	1.41 (0.97-2.03)	0.070	1.48 (1.00-2.19)	0.052
	Long-standing persistent	3,098	147 (4.7)	1.75 (1.33-2.31)	<0.001	1.99 (1.47-2.70)	<0.001
Systolic blood pressure	<130 mm Hg^b^	3,725	133 (3.6)	—		—	
	≥130 mm Hg to <140 mm Hg	1,437	43 (3.0)	0.83 (0.59-1.18)	0.299	0.86 (0.61-1.23)	0.414
	≥140 mm Hg	1,401	76 (5.4)	1.51 (1.14-2.00)	0.004	1.51 (1.12-2.04)	0.007
Severe liver dysfunction	Yes	59	3 (5.1)	1.43 (0.46-4.47)	0.534	1.54 (0.49-4.87)	0.458
	No^b^	7,045	267 (3.8)	—		—	
Diabetes mellitus	Yes (HbA_1c_ < 6.0%)	291	9 (3.1)	0.89 (0.45-1.73)	0.728	0.87 (0.44-1.72)	0.699
	Yes (HbA_1c_ ≥ 6.0%)	1,375	67 (4.9)	1.37 (1.04-1.82)	0.027	1.57 (1.16-2.11)	0.003
	No^b^	5,067	180 (3.6)	—		—	
Hyperuricemia	Yes	1,954	62 (3.2)	0.80 (0.60-1.06)	0.122	0.74 (0.54-1.02)	0.063
	No^b^	5,150	208 (4.0)	—		—	
Heart failure, reduced LVEF	Yes	3,728	139 (3.7)	0.99 (0.78-1.26)	0.929	0.85 (0.65-1.10)	0.211
	No^b^	3,376	131 (3.9)	—		—	
Myocardial infarction	Yes	683	22 (3.2)	0.84 (0.54-1.29)	0.418	0.90 (0.55-1.47)	0.678
	No	6,421	248 (3.9)	—		—	
Cerebrovascular disease	Yes	2,130	114 (5.4)	1.74 (1.36-2.21)	<0.001	1.83 (1.41-2.37)	<0.001
	No^b^	4,974	156 (3.1)	—		—	
Other thromboembolic disease	Yes	818	39 (4.8)	1.31 (0.93-1.84)	0.120	1.32 (0.91-1.91)	0.140
	No^b^	6,286	231 (3.7)	—		—	
Active cancer	Yes	737	22 (3.0)	0.79 (0.51-1.22)	0.287	0.68 (0.42-1.10)	0.115
	No^b^	6,367	248 (3.9)	—		—	
Dementia	Yes	948	38 (4.0)	1.14 (0.81-1.60)	0.465	0.94 (0.66-1.34)	0.738
	No^b^	6,156	232 (3.8)	—		—	
Fall within 1 year	Yes	760	39 (5.1)	1.50 (1.06-2.11)	0.020	1.34 (0.94-1.92)	0.108
	No^b^	5,511	200 (3.6)	—		—	
Catheter ablation	Yes	324	10 (3.1)	0.77 (0.41-1.46)	0.426	1.03 (0.54-1.96)	0.926
	No^b^	6,780	260 (3.8)	—		—	
Antiarrhythmic agents	Yes	4,070	149 (3.7)	0.90 (0.71-1.15)	0.414	0.92 (0.72-1.18)	0.518
	No^b^	3,034	121 (4.0)	—		—	
Proton pump inhibitors	Yes	3,272	116 (3.6)	0.88 (0.69-1.12)	0.302	0.92 (0.71-1.20)	0.540
	No^b^	3,832	154 (4.0)	—		—	
P-glycoprotein inhibitors	Yes	140	2 (1.4) (1.43)	0.38 (0.10-1.54)	0.177	0.22 (0.03-1.56)	0.130
	No^b^	6,964	268 (3.9)	—		—	
Dyslipidemia	Yes	3,189	120 (3.8)	0.96 (0.75-1.22)	0.715	0.95 (0.73-1.24)	0.721
	No^b^	3,915	150 (3.8)	—		—	
Gastrointestinal disease	Yes	2,406	89 (3.7)	0.95 (0.74-1.22)	0.689	0.92 (0.70-1.22)	0.561
	No^b^	4,698	181 (3.9)	—		—	
Antiplatelet agents	Yes (only 1 agent)	3,024	112 (3.7)	0.92 (0.72-1.17)	0.513	0.97 (0.70-1.35)	0.868
	No^b^	4,060	158 (3.9)	—		—	
Polypharmacy	<5 agents^b^	922	30 (3.3)	—		—	
	≥5 agents	6,053	233 (3.9)	1.19 (0.81-1.74)	0.374	1.29 (0.86-1.94)	0.226
Creatinine clearance	≥15 mL/min to <30 mL/min <30 mL/min	2,856	119 (4.2)	1.29 (1.00-1.67)	0.050	1.40 (1.04-1.87)	0.026
	≥30 mL/min^b^	3,398	116 (3.4)	—		—	
Major bleeding							
Total		7,104	198 (2.8)	—		—	
Sex	Male^b^	3,109	90 (2.9)	—		—	
	Female	3,995	108 (2.7)	0.91 (0.69-1.20)	0.513	0.98 (0.69-1.39)	0.901
Body weight	≤45 kg	2,687	73 (2.7)	0.94 (0.70-1.26)	0.678	0.98 (0.67-1.45)	0.933
	>45 kg^b^	4,088	119 (2.9)	—		—	
History of major bleeding	Yes	681	33 (4.9)	1.89 (1.30-2.74)	<0.001	1.62 (1.04-2.53)	0.034
	No^b^	6,423	165 (2.6)	—		—	
Type of AF	Paroxysmal^b^	2,844	73 (2.6)	—		—	
	Persistent	1,162	33 (2.8)	1.14 (0.75-1.72)	0.539	1.01 (0.65-1.55)	0.975
	Long-standing persistent	3,098	92 (3.0)	1.18 (0.87-1.61)	0.281	1.02 (0.73-1.42)	0.911
Systolic blood pressure	<130 mm Hg^b^	3,725	110 (3.0)	—		—	
	≥130 mm Hg to <140 mm Hg	1,437	32 (2.2)	0.75 (0.50-1.11)	0.148	0.80 (0.54-1.20)	0.285
	≥140 mm Hg	1,401	40 (2.9)	0.95 (0.66-1.36)	0.780	1.02 (0.70-1.47)	0.935
Severe liver dysfunction	Yes	59	5 (8.5)	3.46 (1.42-8.40)	0.006	2.90 (1.18-7.12)	0.021
	No^b^	7,045	193 (2.7)	—		—	
Diabetes mellitus	Yes (HbA_1c_ <6.0%)	291	7 (2.4)	0.93 (0.43-1.99)	0.851	0.85 (0.39-1.84)	0.684
	Yes (HbA_1c_ ≥6.0%)	1,375	40 (2.9)	1.10 (0.77-1.57)	0.596	1.14 (0.79-1.66)	0.480
	No^b^	5,067	134 (2.6)	—		—	
Hyperuricemia	Yes	1,954	57 (2.9)	1.09 (0.80-1.48)	0.591	0.96 (0.68-1.34)	0.796
	No^b^	5,150	141 (2.7)	—		—	
Heart failure, reduced LVEF	Yes	3,728	120 (3.2)	1.45 (1.09-1.93)	0.011	1.29 (0.95-1.76)	0.106
	No^b^	3,376	78 (2.3)	—		—	
Myocardial infarction	Yes	683	19 (2.8)	1.01 (0.63-1.61)	0.980	0.98 (0.58-1.66)	0.941
	No^b^	6,421	179 (2.8)	—		—	
Cerebrovascular disease	Yes	2,130	70 (3.3)	1.29 (0.96-1.73)	0.086	1.17 (0.86-1.60)	0.320
	No^b^	4,974	128 (2.6)	—		—	
Other thromboembolic disease	Yes	818	30 (3. 7)	1.40 (0.95-2.06)	0.092	1.35 (0.90-2.03)	0.149
	No^b^	6,286	168 (2.7)	—		—	
Active cancer	Yes	737	28 (3.8)	1.48 (1.00-2.21)	0.053	1.21 (0.79-1.85)	0.385
	No^b^	6,367	170 (2.7)	—		—	
Dementia	Yes	948	27 (2.9)	1.10 (0.73-1.65)	0.643	1.02 (0.67-1.55)	0.926
	No^b^	6,156	171 (2.8)	—		—	
Fall within 1 year	Yes	760	43 (5.7)	2.67 (1.89-3.78)	<0.001	2.26 (1.58-3.24)	<0.001
	No^b^	5,511	125 (2.3)	—		—	
Catheter ablation	Yes	324	11 (3.4)	1.19 (0.65-2.18)	0.578	1.32 (0.71-2.46)	0.385
	No^b^	6,780	187 (2.8)	—		—	
Antiarrhythmic agents	Yes	4,070	104 (2.6)	0.81 (0.61-1.07)	0.144	0.80 (0.60-1.08)	0.143
	No^b^	3,034	94 (3.1)	—		—	
Proton pump inhibitors	Yes	3,272	91 (2.8)	1.00 (0.75-1.32)	0.983	0.95 (0.70-1.28)	0.719
	No^b^	3,832	107 (2.8)	—		—	
P-glycoprotein inhibitors	Yes	140	2 (1.4)	0.53 (0.13-2.13)	0.372	0.56 (0.14-2.28)	0.420
	No^b^	6,964	196 (2.8)	—		—	
Dyslipidemia	Yes	3,189	85 (2.7)	0.90 (0.68-1.19)	0.458	0.90 (0.66-1.23)	0.509
	No^b^	3,915	113 (2.9)	—		—	
Gastrointestinal disease	Yes	2,406	77 (3.2)	1.24 (0.93-1.64)	0.147	1.08 (0.79-1.47)	0.620
	No^b^	4,698	121 (2.6)	—		—	
Antiplatelet agents	Yes (only 1 agent)	3,024	73 (2.4)	0.76 (0.57-1.02)	0.068	0.82 (0.55-1.22)	0.328
	No^b^	4,060	124 (3.1)	—		—	
Polypharmacy	<5 agents^b^	922	21 (2.3)	—		—	
	≥5 agents	6,053	171 (2.8)	1.25 (0.79-1.97)	0.336	1.17 (0.72-1.90)	0.534
Creatinine clearance	≥15 mL/min to <30 mL/min	2,856	82 (2.9)	1.11 (0.82-1.49)	0.492	0.94 (0.66-1.35)	0.752
	≥30 mL/min^b^	3,398	93 (2.7)	—		—	
Intracranial hemorrhage							
Total		7,104	122 (1.7)	—		—	
Sex	Male^b^	3,109	55 (1.8)	—		—	
	Female	3,995	67 (1.7)	0.92 (0.65-1.32)	0.660	0.74 (0.47-1.18)	0.207
Body weight	≤45 kg	2,687	55 (1.8)	1.30 (0.90-1.86)	0.158	1.50 (0.91-2.47)	0.114
	>45 kg^b^	4,088	64 (1.6)	—		—	
History of major bleeding	Yes	681	18 (2.6)	1.62 (0.98-2.67)	0.059	1.40 (0.79-2.47)	0.252
	No^b^	6,423	104 (1.6)	—		—	
Type of AF	Paroxysmal^b^	2,844	45 (1.6)	—		—	
	Persistent	1,162	19 (1.6)	1.06 (0.62-1.82)	0.819	1.04 (0.59-1.83)	0.886
	Long-standing persistent	3,098	58 (1.9)	1.21 (0.82-1.79)	0.333	1.12 (0.73-1.72)	0.594
Systolic blood pressure	<130 mm Hg^b^	3,725	69 (1.9)	—		—	
	≥130 mm Hg to <140 mm Hg	1,437	15 (1.0)	0.56 (0.32-0.98)	0.041	0.61 (0.35-1.07)	0.087
	≥140 mm Hg	1,401	29 (2.1)	1.10 (0.71-1.70)	0.668	1.16 (0.74-1.83)	0.508
Severe liver dysfunction	Yes	59	4 (6.8)	4.42 (1.63-11.97)	0.004	3.55 (1.27-9.89)	0.015
	No^b^	7,045	118 (1.7)	—		—	
Diabetes mellitus	Yes (HbA_1c_ < 6.0%)	291	5 (1.7)	1.06 (0.43-2.61)	0.900	0.95 (0.38-2.37)	0.910
	Yes (HbA_1c_ ≥ 6.0%)	1,375	22 (1.6)	0.96 (0.60-1.54)	0.867	1.04 (0.64-1.71)	0.863
	No^b^	5,067	84 (1.7)	—		—	
Hyperuricemia	Yes	1,954	35 (1.8)	1.09 (0.73-1.61)	0.683	1.04 (0.68-1.61)	0.848
	No^b^	5,150	87 (1.7)	—		—	
Heart failure, reduced LVEF	Yes	3,728	69 (1.9)	1.22 (0.85-1.75)	0.271	1.07 (0.72-1.59)	0.730
	No^b^	3,376	53 (1.6)	—		—	
Myocardial infarction	Yes	683	11 (1.6)	0.94 (0.50-1.74)	0.840	0.89 (0.44-1.81)	0.746
	No^b^	6,421	111 (1.7)	—		—	
Cerebrovascular disease	Yes	2,130	47 (2.2)	1.48 (1.03-2.13)	0.036	1.43 (0.97-2.11)	0.075
	No^b^	4,974	75 (1.5)	—		—	
Other thromboembolic disease	Yes	818	16 (2.0)	1.17 (0.69-1.98)	0.552	1.13 (0.65-1.96)	0.667
	No^b^	6,286	106 (1.7)	—		—	
Active cancer	Yes	737	15 (2.0)	1.26 (0.73-2.16)	0.405	1.03 (0.58-1.83)	0.917
	No^b^	6,367	107 (1.7)	—		—	
Dementia	Yes	948	18 (1.9)	1.21 (0.73-1.99)	0.461	1.07 (0.64-1.81)	0.788
	No^b^	6,156	104 (1.7)	—		—	
Fall within 1 year	Yes	760	29 (3.8)	3.08 (2.00-4.74)	<0.001	2.61 (1.66-4.11)	<0.001
	No^b^	5,511	73 (1.3)	—		—	
Catheter ablation	Yes	324	8 (2.5)	1.41 (0.69-2.90)	0.343	1.63 (0.78-3.43)	0.196
	No^b^	6,780	114 (1.7)	—		—	
Antiarrhythmic agents	Yes	4,070	66 (1.6)	0.87 (0.61-1.24)	0.429	0.92 (0.63-1.34)	0.657
	No^b^	3,034	56 (1.9)	—		—	
Proton pump inhibitors	Yes	3,272	44 (1.3)	0.66 (0.46-0.95)	0.027	0.66 (0.45-0.99)	0.045
	No^b^	3,832	78 (2.0)	—		—	
P-glycoprotein inhibitors	Yes	140	2 (1.4)	0.87 (0.22-3.52)	0.845	0.92 (0.23-3.78)	0.911
	No^b^	6,964	120 (1.7)	—		—	
Dyslipidemia	Yes	3,189	56 (1.8)	1.01 (0.71-1.45)	0.943	1.12 (0.76-1.67)	0.558
	No^b^	3,915	66 (1.7)	—		—	
Gastrointestinal disease	Yes	2,406	45 (1.9)	1.13 (0.78-1.63)	0.511	1.04 (0.69-1.55)	0.857
	No^b^	4,698	77 (1.6)	—		—	
Antiplatelet agents	Yes (only 1 agent)	3,024	44 (1.5)	0.74 (0.51-1.07)	0.112	0.83 (0.50-1.38)	0.471
	No^b^	4,060	77 (1.9)	—		—	
Polypharmacy	<5 agents^b^	922	18 (2.0)	—		—	
	≥5 agents	6,053	98 (1.6)	0.83 (0.50-1.37)	0.470	0.88 (0.51-1.53)	0.654
Creatinine clearance	≥15 mL/min to <30 mL/min <30 mL/min	2,856	47 (1.7)	1.02 (0.70-1.50)	0.912	0.87 (0.56-1.37)	0.558
	≥30 mL/min^b^	3,398	58 (1.7)	—		—	

HbA_1c_ = glycosylated hemoglobin; LVEF = left ventricular ejection fraction; other abbreviations as in Table 1.

a Sex; body weight; history of bleeding; type of AF; systolic blood pressure; severe liver dysfunction; diabetes mellitus; hyperuricemia; heart failure and/or reduced LVEF; myocardial infarction; cerebrovascular disease; other thromboembolic disease; active cancer; dementia; fall within 1 year; history of catheter ablation; dyslipidemia; creatinine clearance; gastrointestinal diseases; polypharmacy (5 or more); anticoagulant agent; and use of antiarrhythmic agents, proton pump inhibitors, P-glycoprotein inhibitors, and antiplatelet agents were included as an adjustment factor in the model. Type of anticoagulant agents were included in the multivariate analysis model as an explanatory factor.

b Reference.

Additionally, we calculated the HRs for outcomes in the reference group (ie, the non–high-risk group) according to baseline variables (Supplemental Table 1). AF type in stroke/SE, falls in major bleeding, and severe liver dysfunction and falls in intracranial hemorrhage showed a significant difference in HRs, even in the reference group; however, the HR value was larger in the high-risk group. In the high-risk group, cerebrovascular disease was the only factor that was not found to be a specific risk factor for stroke/SE.

## Discussion

Until now, real-world evidence on the effectiveness and safety of OAC agents in very elderly NVAF patients with high bleeding risk has been scarce. In the present subanalysis, we categorized ANAFIE patients as having high bleeding risk by using the inclusion criteria of the ELDERCARE-AF trial,^9^ consisting of age of ≥80 years; CHADS_2_ score of ≥2; and the presence of ≥1 bleeding risk factor, including severe renal impairment, prior bleeding history at critical sites, low body weight, and continuous antiplatelet use. It is well established that these factors are associated with increased bleeding incidence during OAC therapy. Importantly, patients with severe renal impairment and prior bleeding history at critical sites were excluded from landmark DOAC agent trials, such as RE-LY (Randomized Evaluation of Long-Term Anticoagulation Therapy),^15^ ROCKET-AF (Rivaroxaban Once Daily Oral Direct Factor Xa Inhibition Compared with Vitamin K Antagonism for Prevention of Stroke and Embolism Trial in Atrial Fibrillation),^16^ ARISTOTLE (Apixaban for Reduction in Stroke and Other Thromboembolic Events in Atrial Fibrillation),^17^ and ENGAGE-AF TIMI 48 (Effective Anticoagulation with Factor Xa Next Generation in Atrial Fibrillation–Thrombolysis In Myocardial Infarction 48).^18^ Thus, we deemed it necessary to evaluate the real-world rate of OAC agent use and the outcomes during OAC treatment among elderly Japanese NVAF patients who had high bleeding risk from the ANAFIE registry.

This subanalysis of the ANAFIE registry suggests the usefulness of DOAC agents compared with warfarin and with no OAC agent use in clinical practice in elderly patients at high bleeding risk. First, we found that 22.0% of the ANAFIE patients met the inclusion criteria of the ELDERCARE-AF trial,^9^ and this high-risk group of patients had high rates of OAC use (89.0% vs 93.4% in the reference group), with more than a half (58.9%) receiving DOAC agents (vs 69.1% in the reference group). Among the high-risk patients who received DOAC agents, most of the high-risk patients received on-label reduced doses of DOAC agents (71.5%), with a minor proportion receiving off-label low doses (5.9%). It was previously reported that the risk of intracranial hemorrhage during warfarin therapy was higher among Asian patients than White patients,^19^ which may explain the tendency of Japanese physicians to prescribe DOAC agents for stroke prevention, even for elderly NVAF patients with high bleeding risk. Of note, the prescribed DOAC doses were appropriately reduced in most cases, whereas the mean TTR during warfarin therapy was 71%. Thus, the prescription of DOAC agents at the doses indicated in the package insert was confirmed for many of the ANAFIE patients who were considered appropriate candidates for OAC therapy, despite the high bleeding risk.

Second, we found that all the events occurred at higher rates in the high-risk group compared with the reference group. This was not surprising because the high-risk group included patients who were older, had more comorbidities, and were at higher risk for stroke and major bleeding.

Third, in the high-risk group, we clearly showed by the multivariate analysis that compared with the nonuse of OAC agents, DOAC agent use was associated with reduced incidences of stroke/SE and all-cause death without significantly increasing major bleeding. Furthermore, the effectiveness and safety of DOAC agents were superior to those of warfarin in reducing stroke/SE, major bleeding, and intracranial hemorrhage. The HR (0.88) of all-cause death was lower with DOAC agents compared with warfarin use, but the difference was not significant. These results were consistent with those seen in the overall ANAFIE registry population.^10^ It should be pointed out that the rate for all bleeding events (major bleeding, clinically relevant bleeding, and minor bleeding) was significantly higher in the DOAC agent use than in the nonuse-of-OAC-agents group. A careful observation should be required even when DOAC agents are selected in high-risk group patients.

For the higher incidence of major bleeding in the high-risk group than in the reference group, as expected, multivariate analysis for the baseline characteristics of high-risk patients revealed that a history of major bleeding, severe liver dysfunction, and falls within 1 year were significant independent predictors for major bleeding. Based on these findings, we suggest that careful observation for the occurrence of major bleeding should be required for the management of elderly high-risk patients with these clinical characteristics when OAC agent use is considered.

The ELDERCARE-AF trial evaluated the efficacy and safety of very-low-dose edoxaban (15 mg) vs placebo and demonstrated that edoxaban was superior to placebo for preventing stroke/SE without a significant increase in major bleeding events.^9^ It should be noted that all ELDERCARE-AF patients were considered inappropriate for standard OAC therapy at approved doses.^9^ In fact, 57% of the patients had not received any OAC therapy before participating in the trial. In contrast, the remaining patients had been previously treated with OAC agents but discontinued DOAC agents or were treated with warfarin at an insufficient control level. The present subanalysis of the ANAFIE registry defined patients with similar characteristics to patients included in the ELDERCARE-AF trial as the high-risk group. However, considering that 58.9% of these patients received DOAC agents and most of them were prescribed on-label reduced DOAC agent doses at study entry, the risk of bleeding might have been lower among those patients in the present analysis than among patients in the ELDERCARE-AF trial.^9^ Further studies are needed to evaluate the use of OAC therapy, including very-low-dose edoxaban in the patient group in which no OAC agent was used, presumably because of the extremely high bleeding risk.

### Study limitations

The main limitation of the present analysis is that it is a post hoc evaluation. Additionally, in this subanalysis, patients with a high risk of bleeding were grouped according to the definition used in the ELDERCARE-AF trial, but the mean HAS-BLED score in the high-risk group of the ANAFIE registry was 2.2. Thus, the high bleeding risk in the high-risk group was not linked to the HAS-BLED score. Furthermore, patients with a CrCl of <15 mL/min or undergoing dialysis were not included in the high-risk group for bleeding in this study to verify the significance of DOAC agents because they are not indicated for DOAC agents. Therefore, this may have affected the power of the analysis. Because the ANAFIE registry enrolled patients eligible for OAC therapy, comparisons with the data from the ELDERCARE-AF trial, which enrolled patients who were not eligible for standard OAC therapy at approved doses, are limited. Although many high-risk group patients prescribed with DOAC agents (76%) received appropriate on-label doses, other patients (13%) received inappropriately reduced off-label doses while showing similar 2-year incidences of stroke/SE, major bleeding, and all-cause mortality. Additional limitations of the ANAFIE registry have been reported previously^10^ and include the enrollment of only Japanese NVAF patients; the fact that variables such as changes in OAC agent during follow-up were not considered; the fact that TTR for warfarin was reported at baseline but was not re-evaluated during the follow-up; and the fact that the study did not limit the inclusion of patients who had already been receiving anticoagulant treatment before enrollment. Finally, outcomes for warfarin-treated patients were not reported according to individual TTR groups (ie, <40%, 40% to <60%, 60% to <80%, and ≥80%) or international normalized ratio, and we are unable to speculate as to how this may have affected the results observed.

## Conclusions

Patients from the ANAFIE registry at high bleeding risk had higher incidences of stroke/SE, major bleeding, intracranial hemorrhage, gastrointestinal bleeding, cardiovascular events, and all-cause death than patients in the reference group, despite a high prevalence of OAC therapy (89.0%). Our data indicate that DOAC agent use was associated with reduced risks without significantly increasing major bleeding events compared with nonuse of OAC agents in elderly NVAF patients at high bleeding risk. The present results also indicate that DOAC agent use was superior to warfarin in reducing stroke/SE, major bleeding, and intracranial hemorrhage in these patients.

Perspectives **COMPETENCY IN MEDICAL KNOWLEDGE:** Our findings indicate that, compared with nonuse of oral anticoagulant agents, DOAC agent use may be associated with reduced risks without significantly increasing major bleeding events among elderly nonvalvular atrial fibrillation patients at high bleeding risk.

**TRANSLATIONAL OUTLOOK:** Further studies are warranted to confirm that DOAC agent use is superior to warfarin for reducing stroke/systemic embolism, major bleeding, and intracranial hemorrhage in elderly patients with nonvalvular atrial fibrillation and high bleeding risk.

## Funding Support and Author Disclosures

This research was supported by Daiichi Sankyo Co, Ltd. Dr Okumura received remuneration from Nippon Boehringer Ingelheim, Daiichi Sankyo, Johnson & Johnson, and Medtronic. Dr Yamashita has received research funding from Bristol-Myers Squibb, Bayer, and Daiichi Sankyo; manuscript fees from Daiichi Sankyo and Bristol-Myers Squibb; and remuneration from Daiichi Sankyo, Bayer, Pfizer Japan, Bristol-Myers Squibb, and Ono Pharmaceutical. Dr Akao has received research funding from Bayer and Daiichi Sankyo and remuneration from Bristol-Myers Squibb, Nippon Boehringer Ingelheim, Bayer, and Daiichi Sankyo. Dr Atarashi has received remuneration from Daiichi Sankyo. Dr Ikeda has received research grants from Daiichi Sankyo, Medtronic Japan, and Japan Lifeline; has received honoraria from Ono Pharma, Bayer Yakuhin, Daiichi Sankyo, Bristol-Myers Squibb, and Pfizer; and was a member of the Advisory Board for Bayer Yakuhin, Bristol-Myers Squibb, and Daiichi Sankyo. Dr Koretsune has received remuneration from Daiichi Sankyo, Bayer, and Nippon Boehringer Ingelheim. Dr Shimizu has received research funding from Bristol-Myers Squibb, Daiichi Sankyo, and Nippon Boehringer Ingelheim and patent royalties/licensing fees from Daiichi Sankyo, Pfizer Japan, Bristol-Myers Squibb, Bayer, and Nippon Boehringer Ingelheim. Dr Suzuki has received remuneration from Bristol-Myers Squibb and Daiichi Sankyo. Dr Tsutsui has received research funding from Daiichi Sankyo, Mitsubishi Tanabe Pharma, Nippon Boehringer Ingelheim, and IQVA Services Japan; remuneration from Daiichi Sankyo, Bayer, Nippon Boehringer Ingelheim, Pfizer Japan, Otsuka Pharmaceutical, and Mitsubishi Tanabe Pharma; scholarship funding from Daiichi Sankyo, Mitsubishi Tanabe Pharma, and Teijin Pharma; and consultancy fees from Novartis Pharma, Pfizer Japan, Bayer, Nippon Boehringer Ingelheim, and Ono Pharmaceutical. Dr Toyoda has received honoraria from Daiichi Sankyo, Bayer, Bristol-Myers Squibb, and Takeda. Dr Hirayama participated in a course endowed by Boston Scientific Japan; has received research funding from Daiichi Sankyo and Bayer; has received remuneration from Bayer, Daiichi Sankyo, Bristol-Myers Squibb, Nippon Boehringer Ingelheim, Sanofi, Astellas Pharma, Sumitomo Dainippon Pharma, Amgen Astellas BioPharma, and AstraZeneca; and has received patent royalties/licensing fees from Toa Eiyo M. Dr Yasaka received research funding from Nippon Boehringer Ingelheim and remuneration from Nippon Boehringer Ingelheim, Daiichi Sankyo, Bayer, Bristol-Myers Squibb, Pfizer Japan, and CSL Behring. Dr Yamaguchi acted as an Advisory Board member of Daiichi Sankyo; and has received honoraria from Daiichi Sankyo and Bristol-Myers Squibb. Dr Teramukai has received research funding from Nippon Boehringer Ingelheim and remuneration from Daiichi Sankyo, Sanofi, Takeda, Chugai Pharmaceutical, Solasia Pharma, Bayer, Sysmex, Nipro, NapaJen Pharma, Gunze, and Atworking. Dr Kimura has stock and is an employee of Daiichi Sankyo. Dr Morishima and Atsushi Takita are employees of Daiichi Sankyo. Dr Inoue has received honoraria from Daiichi Sankyo, Bayer, Bristol-Myers Squibb and consultancy fees from Daiichi Sankyo.
