# Supplementary material for: Oral Anticoagulants in Very Elderly Nonvalvular Atrial Fibrillation Patients With High Bleeding Risks: ANAFIE Registry
Source: JACC Asia. 2022 Nov 15;2(6):720–33. doi: 10.1016/j.jacasi.2022.07.008 (PMC9700004; doi:10.1016/j.jacasi.2022.07.008)
Supplement: Supplemental Table 1 [file mmc1.docx]

**Oral Anticoagulants in Asian, Very Elderly Non-Valvular Atrial Fibrillation Patients with High-Bleeding Risks: ANAFIE Registry**

**Running title:** OACs in NVAF and high bleeding risk

Ken Okumura, MD, PhD,^a^* Takeshi Yamashita, MD, PhD,^b^ Masaharu Akao, MD, PhD,^c^ Hirotsugu Atarashi, MD, PhD,^d^ Takanori Ikeda, MD, PhD,^e^ Yukihiro Kitsune, MD, PhD,^f^ Wataru Shimizu, MD, PhD,^g^ Shinya Suzuki, MD, PhD,^b^ Hiroyuki Tsutsui, MD, PhD,^c^ Kazunori Toyoda, MD, PhD,^c^ Atsushi Hirayama, MD, PhD,^j^ Masahiro Yasaka, MD, PhD,^k^ Takenori Yamaguchi, MD, PhD,^i^ Satoshi Teramukai, PhD,^l^ Tetsuya Kimura, MS,^m^ Yoshiyuki Morishima, PhD,^m^ Atsushi Takita, MS,^n^ and Hiroshi Inoue, MD, PhD,^o^

**Supplemental Table 1. Univariate and multivariate analyses of outcomes according to baseline variables in the Reference group (non-high-risk group)**

| **Outcomes and factors** | **Variables** | **N** | **Event (%)** | **Univariate** **analysis** | | **Multivariate analysis^#^** | |  |
| --- | --- | --- | --- | --- | --- | --- | --- | --- |
|  |  |  |  | **HR (95% CI)** | ***P* value** | **HR (95% CI)** | ***P* value** |  |
| **Stroke/systemic embolism** | | | | | | | | |
| Total |  | 25,171 | 700 (2.8) | － |  | － |  |  |
| Sex | Male^*^ | 15,373 | 455 (3.0) | － |  | － |  |  |
|  | Female | 9,798 | 245 (2.5) | 0.84 (0.72, 0.98) | 0.025 | 0.96 (0.79, 1.15) | 0.641 |  |
| Body weight | ≤45 kg | 1,306 | 28 (2.1) | 0.81 (0.55, 1.18) | 0.264 | 0.84 (0.54, 1.33) | 0.462 |  |
|  | >45 kg^*^ | 21,368 | 587 (2.7) | － |  | － |  |  |
| History of major bleeding | Yes | 518 | 24 (4.6) | 1.75 (1.17, 2.63) | 0.007 | 1.32 (0.86, 2.01) | 0.206 |  |
|  | No^*^ | 24,653 | 676 (2.7) | － |  | － |  |  |
| Type of AF | Paroxysmal^*^ | 10,742 | 206 (1.9) | － |  | － |  |  |
|  | Persistent | 4,174 | 146 (3.5) | 1.86 (1.51, 2.30) | <0.001 | 1.63 (1.28, 2.08) | <0.001 |  |
|  | Long-standing persistent | 10,255 | 348 (3.4) | 1.81 (1.52, 2.15) | <0.001 | 1.61 (1.31, 1.96) | <0.001 |  |
| Systolic blood pressure | <130 mmHg^*^ | 12,493 | 324 (2.6) | － |  | － |  |  |
|  | ≥130 mmHg to  <140 mmHg | 5,500 | 161 (2.9) | 1.12 (0.93, 1.36) | 0.223 | 1.15 (0.94, 1.42) | 0.175 |  |
|  | ≥140 mmHg | 5,127 | 151 (2.9) | 1.13 (0.93, 1.37) | 0.220 | 1.20 (0.97, 1.48) | 0.094 |  |
| Severe liver dysfunction | Yes | 236 | 11 (4.7) | 1.76 (0.97, 3.19) | 0.063 | 1.80 (0.96, 3.38) | 0.068 |  |
|  | No^*^ | 24,935 | 689 (2.8) | － |  | － |  |  |
| Diabetes mellitus | Yes (HbA1c <6.0%) | 968 | 36 (3.7) | 1.43 (1.02, 2.01) | 0.038 | 1.03 (0.69, 1.54) | 0.894 |  |
|  | Yes (HbA1c ≥6.0%) | 4,449 | 138 (3.1) | 1.18 (0.98, 1.43) | 0.085 | 1.06 (0.85, 1.31) | 0.604 |  |
|  | No^*^ | 18,475 | 487 (2.6) | － |  | － |  |  |
| Hyperuricemia | Yes | 5,360 | 164 (3.1) | 1.15 (0.96, 1.37) | 0.119 | 1.04 (0.85, 1.27) | 0.731 |  |
|  | No^*^ | 19,811 | 536 (2.7) | － |  | － |  |  |
| Heart failure, reduced LVEF | Yes | 8,549 | 261 (3.1) | 1.18 (1.01, 1.38) | 0.034 | 0.92 (0.77, 1.10) | 0.358 |  |
|  | No^*^ | 16,622 | 439 (2.6) | － |  | － |  |  |
| Myocardial infarction | Yes | 1,168 | 42 (3.6) | 1.35 (0.99, 1.85) | 0.058 | 1.48 (1.03, 2.15) | 0.036 |  |
|  | No^*^ | 24,003 | 658 (2.7) | － |  | － |  |  |
| Cerebrovascular disease | Yes | 5,173 | 284 (5.5) | 2.72 (2.34, 3.16) | <0.001 | 2.53 (2.12, 3.01) | <0.001 |  |
|  | No^*^ | 19,998 | 416 (2.1) | － |  | － |  |  |
| Other thromboembolic disease | Yes | 1,967 | 90 (4.6) | 1.76 (1.41, 2.20) | <0.001 | 1.46 (1.12, 1.89) | 0.004 |  |
|  | No^*^ | 23,204 | 610 (2.6) | － |  | － |  |  |
| Active cancer | Yes | 2,832 | 88 (3.1) | 1.17 (0.93, 1.46) | 0.180 | 1.14 (0.89, 1.46) | 0.285 |  |
|  | No^*^ | 22,339 | 612 (2.7) | － |  | － |  |  |
| Dementia | Yes | 1,564 | 69 (4.4) | 1.76 (1.37, 2.26) | <0.001 | 1.30 (0.98, 1.73) | 0.071 |  |
|  | No^*^ | 23,607 | 631 (2.7) | － |  | － |  |  |
| Fall within 1 year | Yes | 1,587 | 69 (4.3) | 1.73 (1.34, 2.22) | <0.001 | 1.40 (1.06, 1.85) | 0.019 |  |
|  | No^*^ | 20,806 | 541 (2.6) | － |  | － |  |  |
| Catheter ablation | Yes | 2,646 | 32 (1.2) | 0.40 (0.28, 0.56) | <0.001 | 0.43 (0.29, 0.65) | <0.001 |  |
|  | No^*^ | 22,525 | 668 (3.0) | － |  | － |  |  |
| Antiarrhythmic agents | Yes | 14,064 | 364 (2.6) | 0.85 (0.73, 0.99) | 0.035 | 1.01 (0.85, 1.20) | 0.897 |  |
|  | No^*^ | 11,107 | 336 (3.0) | － |  | － |  |  |
| Proton pump inhibitors | Yes | 8,555 | 217 (2.5) | 0.87 (0.74, 1.03) | 0.099 | 0.92 (0.76, 1.11) | 0.394 |  |
|  | No^*^ | 16,616 | 483 (2.9) | － |  | － |  |  |
| P-glycoprotein inhibitors | Yes | 421 | 15 (3.6) | 1.30 (0.78, 2.17) | 0.313 | 1.32 (0.74, 2.34) | 0.350 |  |
|  | No^*^ | 24,750 | 685 (2.8) | － |  | － |  |  |
| Dyslipidemia | Yes | 10,539 | 276 (2.6) | 0.89 (0.77, 1.04) | 0.141 | 0.87 (0.73, 1.04) | 0.129 |  |
|  | No^*^ | 14,632 | 424 (2.9) | － |  | － |  |  |
| Gastrointestinal disease | Yes | 7,061 | 203 (2.9) | 1.04 (0.89, 1.23) | 0.607 | 0.92 (0.76, 1.12) | 0.411 |  |
|  | No^*^ | 18,110 | 497 (2.7) | － |  | － |  |  |
| Antiplatelet agents | Yes (only one) agent) | 2,250 | 51 (2.3) | 0.82 (0.62, 1.09) | 0.171 | 0.72 (0.53, 0.99) | 0.043 |  |
|  | No^*^ | 22,281 | 622 (2.8) | － |  | － |  |  |
| Polypharmacy | <5 agents^*^ | 7,510 | 202 (2.7) | － |  | － |  |  |
|  | ≥5 agents | 16,934 | 473 (2.8) | 1.06 (0.89, 1.24) | 0.523 | 0.95 (0.78, 1.16) | 0.599 |  |
| Creatinine clearance | ≥15 mL/min to  <30 mL/min <30 mL/min | 618 | 20 (3.2) | 1.26 (0.81, 1.97) | 0.311 | 1.09 (0.65, 1.82) | 0.738 |  |
|  | ≥30 mL/min^*^ | 18,941 | 512 (2.7) | － |  | － |  |  |
| **Major bleeding** | | | | | | | | |
| Total |  | 25,171 | 447 (1.8) | － |  | － |  |  |
| Sex | Male^*^ | 15,373 | 291 (1.9) | － |  | － |  |  |
|  | Female | 9,798 | 156 (1.6) | 0.83 (0.69, 1.01) | 0.066 | 0.79 (0.62, 1.00) | 0.048 |  |
| Body weight | ≤45 kg | 1,306 | 23 (1.8) | 1.05 (0.69, 1.59) | 0.837 | 0.90 (0.52, 1.55) | 0.701 |  |
|  | >45 kg^*^ | 21,368 | 374 (1.8) | － |  | － |  |  |
| History of major bleeding | Yes | 518 | 14 (2.7) | 1.58 (0.93, 2.70) | 0.090 | 1.38 (0.77, 2.49) | 0.279 |  |
|  | No^*^ | 24,653 | 433 (1.8) | － |  | － |  |  |
| Type of AF | Paroxysmal^*^ | 10,742 | 176 (1.6) | － |  | － |  |  |
|  | Persistent | 4,174 | 78 (1.9) | 1.16 (0.89, 1.51) | 0.284 | 1.03 (0.76, 1.40) | 0.825 |  |
|  | Long-standing persistent | 10,255 | 193 (1.9) | 1.17 (0.95, 1.43) | 0.138 | 0.99 (0.78, 1.26) | 0.960 |  |
| Systolic blood pressure | <130 mmHg^*^ | 12,493 | 220 (1.8) | － |  | － |  |  |
|  | ≥130 mmHg to  <140 mmHg | 5,500 | 95 (1.7) | 0.98 (0.77, 1.24) | 0.844 | 1.04 (0.80, 1.35) | 0.785 |  |
|  | ≥140 mmHg | 5,127 | 100 (2.0) | 1.10 (0.87, 1.39) | 0.427 | 1.27 (0.98, 1.64) | 0.072 |  |
| Severe liver dysfunction | Yes | 236 | 9 (3.8) | 2.27 (1.18, 4.40) | 0.015 | 1.99 (0.94, 4.22) | 0.074 |  |
|  | No^*^ | 24,935 | 438 (1.8) | － |  | － |  |  |
| Diabetes mellitus | Yes (HbA1c <6.0%) | 968 | 19 (2.0) | 1.15 (0.73, 1.83) | 0.544 | 0.90 (0.52, 1.55) | 0.704 |  |
|  | Yes (HbA1c ≥6.0%) | 4,449 | 79 (1.8) | 1.04 (0.81, 1.33) | 0.769 | 0.88 (0.67, 1.16) | 0.364 |  |
|  | No^*^ | 18,475 | 317 (1.7) | － |  | － |  |  |
| Hyperuricemia | Yes | 5,360 | 112 (2.1) | 1.25 (1.01, 1.55) | 0.038 | 1.02 (0.79, 1.31) | 0.888 |  |
|  | No^*^ | 19,811 | 335 (1.7) | － |  | － |  |  |
| Heart failure, reduced LVEF | Yes | 8,549 | 167 (2.0) | 1.18 (0.98, 1.43) | 0.086 | 0.94 (0.75, 1.18) | 0.580 |  |
|  | No^*^ | 16,622 | 280 (1.7) | － |  | － |  |  |
| Myocardial infarction | Yes | 1,168 | 26 (2.2) | 1.30 (0.88, 1.94) | 0.190 | 1.05 (0.64, 1.73) | 0.845 |  |
|  | No^*^ | 24,003 | 421 (1.8) | － |  | － |  |  |
| Cerebrovascular disease | Yes | 5,173 | 123 (2.4) | 1.49 (1.21, 1.84) | <0.001 | 1.25 (0.99, 1.60) | 0.066 |  |
|  | No^*^ | 19,998 | 324 (1.6) | － |  | － |  |  |
| Other thromboembolic disease | Yes | 1,967 | 46 (2.3) | 1.37 (1.01, 1.85) | 0.045 | 1.14 (0.79, 1.63) | 0.490 |  |
|  | No^*^ | 23,204 | 401 (1.7) | － |  | － |  |  |
| Active cancer | Yes | 2,832 | 68 (2.4) | 1.46 (1.12, 1.88) | 0.004 | 1.26 (0.93, 1.69) | 0.134 |  |
|  | No^*^ | 22,339 | 379 (1.7) | － |  | － |  |  |
| Dementia | Yes | 1,564 | 36 (2.3) | 1.40 (1.00, 1.97) | 0.053 | 1.29 (0.88, 1.89) | 0.184 |  |
|  | No^*^ | 23,607 | 411 (1.7) | － |  | － |  |  |
| Fall within 1 year | Yes | 1,587 | 49 (3.1) | 1.91 (1.42, 2.58) | <0.001 | 1.64 (1.17, 2.31) | 0.004 |  |
|  | No^*^ | 20,806 | 346 (1.7) | － |  | － |  |  |
| Catheter ablation | Yes | 2,646 | 25 (0.9) | 0.49 (0.33, 0.74) | 0.001 | 0.56 (0.36, 0.86) | 0.009 |  |
|  | No^*^ | 22,525 | 422 (1.9) | － |  | － |  |  |
| Antiarrhythmic agents | Yes | 14,064 | 232 (1.6) | 0.85 (0.71, 1.02) | 0.085 | 0.88 (0.71, 1.08) | 0.222 |  |
|  | No^*^ | 11,107 | 215 (1.9) | － |  | － |  |  |
| Proton pump inhibitors | Yes | 8,555 | 157 (1.8) | 1.06 (0.87, 1.28) | 0.576 | 1.12 (0.89, 1.41) | 0.335 |  |
|  | No^*^ | 16,616 | 290 (1.7) | － |  | － |  |  |
| P-glycoprotein inhibitors | Yes | 421 | 7 (1.7) | 0.94 (0.45, 1.98) | 0.870 | 0.97 (0.43, 2.18) | 0.937 |  |
|  | No^*^ | 24,750 | 440 (1.8) | － |  | － |  |  |
| Dyslipidemia | Yes | 10,539 | 183 (1.7) | 0.95 (0.79, 1.15) | 0.605 | 0.96 (0.77, 1.19) | 0.693 |  |
|  | No^*^ | 14,632 | 264 (1.8) | － |  | － |  |  |
| Gastrointestinal disease | Yes | 7,061 | 125 (1.8) | 0.99 (0.81, 1.22) | 0.935 | 0.89 (0.70, 1.13) | 0.346 |  |
|  | No^*^ | 18,110 | 322 (1.8) | － |  | － |  |  |
| Antiplatelet agents | Yes (only one) agent) | 2,250 | 41 (1.8) | 1.04 (0.76, 1.44) | 0.790 | 0.91 (0.63, 1.32) | 0.630 |  |
|  | No^*^ | 22,281 | 393 (1.8) | － |  | － |  |  |
| Polypharmacy | <5 agents^*^ | 7,510 | 107 (1.4) | － |  | － |  |  |
|  | ≥5 agents | 16,934 | 328 (1.9) | 1.38 (1.11, 1.72) | 0.004 | 1.28 (0.99, 1.66) | 0.061 |  |
| Creatinine clearance | ≥15 mL/min to  <30 mL/min | 618 | 16 (2.6) | 1.57 (0.95, 2.59) | 0.080 | 1.34 (0.77, 2.34) | 0.303 |  |
|  | ≥30 mL/min^*^ | 18,941 | 331 (1.7) | － |  | － |  |  |
| **Intracranial hemorrhage** | | | | | | | | |
| Total |  | 25,171 | 331 (1.3) | － |  | － |  |  |
| Sex | Male^*^ | 15,373 | 220 (1.4) | － |  | － |  |  |
|  | Female | 9,798 | 111 (1.1) | 0.78 (0.62, 0.98) | 0.036 | 0.76 (0.58, 1.00) | 0.054 |  |
| Body weight | ≤45 kg | 1,306 | 15 (1.1) | 0.92 (0.55, 1.55) | 0.753 | 0.93 (0.49, 1.74) | 0.811 |  |
|  | >45 kg^*^ | 21,368 | 277 (1.3) | － |  | － |  |  |
| History of major bleeding | Yes | 518 | 9 (1.7) | 1.37 (0.71, 2.66) | 0.351 | 1.21 (0.59, 2.47) | 0.609 |  |
|  | No^*^ | 24,653 | 322 (1.3) | － |  | － |  |  |
| Type of AF | Paroxysmal^*^ | 10,742 | 130 (1.2) | － |  | － |  |  |
|  | Persistent | 4,174 | 60 (1.4) | 1.21 (0.89, 1.64) | 0.230 | 1.09 (0.77, 1.54) | 0.636 |  |
|  | Long-standing persistent | 10,255 | 141 (1.4) | 1.15 (0.91, 1.47) | 0.237 | 1.02 (0.78, 1.35) | 0.875 |  |
| Systolic blood pressure | <130 mmHg^*^ | 12,493 | 155 (1.2) | － |  | － |  |  |
|  | ≥130 mmHg to <140 mmHg | 5,500 | 73 (1.3) | 1.07 (0.81, 1.41) | 0.657 | 1.08 (0.80, 1.47) | 0.616 |  |
|  | ≥140 mmHg | 5,127 | 78 (1.5) | 1.22 (0.93, 1.60) | 0.154 | 1.41 (1.05, 1.89) | 0.022 |  |
| Severe liver dysfunction | Yes | 236 | 7 (3.0) | 2.38 (1.13, 5.03) | 0.023 | 2.46 (1.09, 5.55) | 0.031 |  |
|  | No^*^ | 24,935 | 324 (1.3) | － |  | － |  |  |
| Diabetes mellitus | Yes (HbA1c <6.0%) | 968 | 11 (1.1) | 0.90 (0.49, 1.66) | 0.746 | 0.84 (0.42, 1.64) | 0.602 |  |
|  | Yes (HbA1c ≥6.0%) | 4,449 | 64 (1.4) | 1.14 (0.86, 1.50) | 0.357 | 1.01 (0.74, 1.38) | 0.928 |  |
|  | No^*^ | 18,475 | 234 (1.3) | － |  | － |  |  |
| Hyperuricemia | Yes | 5,360 | 81 (1.5) | 1.22 (0.95, 1.56) | 0.128 | 1.03 (0.77, 1.38) | 0.841 |  |
|  | No^*^ | 19,811 | 250 (1.3) | － |  | － |  |  |
| Heart failure, reduced LVEF | Yes | 8,549 | 114 (1.3) | 1.04 (0.83, 1.31) | 0.728 | 0.85 (0.65, 1.11) | 0.222 |  |
|  | No^*^ | 16,622 | 217 (1.3) | － |  | － |  |  |
| Myocardial infarction | Yes | 1,168 | 18 (1.5) | 1.21 (0.75, 1.95) | 0.429 | 1.01 (0.56, 1.82) | 0.962 |  |
|  | No^*^ | 24,003 | 313 (1.3) | － |  | － |  |  |
| Cerebrovascular disease | Yes | 5,173 | 95 (1.8) | 1.58 (1.25, 2.01) | <0.001 | 1.36 (1.03, 1.79) | 0.028 |  |
|  | No^*^ | 19,998 | 236 (1.2) | － |  | － |  |  |
| Other thromboembolic disease | Yes | 1,967 | 29 (1.5) | 1.14 (0.78, 1.67) | 0.498 | 1.05 (0.68, 1.61) | 0.837 |  |
|  | No^*^ | 23,204 | 302 (1.3) | － |  | － |  |  |
| Active cancer | Yes | 2,832 | 45 (1.6) | 1.28 (0.93, 1.75) | 0.129 | 1.21 (0.85, 1.73) | 0.278 |  |
|  | No^*^ | 22,339 | 286 (1.3) | － |  | － |  |  |
| Dementia | Yes | 1,564 | 30 (1.9) | 1.60 (1.10, 2.32) | 0.015 | 1.39 (0.91, 2.13) | 0.131 |  |
|  | No^*^ | 23,607 | 301 (1.3) | － |  | － |  |  |
| Fall within 1 year | Yes | 1,587 | 43 (2.7) | 2.29 (1.66, 3.17) | <0.001 | 1.86 (1.28, 2.69) | 0.001 |  |
|  | No^*^ | 20,806 | 254 (1.2) | － |  | － |  |  |
| Catheter ablation | Yes | 2,646 | 20 (0.8) | 0.53 (0.34, 0.84) | 0.006 | 0.64 (0.40, 1.04) | 0.074 |  |
|  | No^*^ | 22,525 | 311 (1.4) | － |  | － |  |  |
| Antiarrhythmic agents | Yes | 14,064 | 168 (1.2) | 0.81 (0.65, 1.01) | 0.057 | 0.90 (0.70, 1.14) | 0.384 |  |
|  | No^*^ | 11,107 | 163 (1.5) | － |  | － |  |  |
| Proton pump inhibitors | Yes | 8,555 | 113 (1.3) | 1.01 (0.81, 1.27) | 0.928 | 1.17 (0.90, 1.52) | 0.236 |  |
|  | No^*^ | 16,616 | 218 (1.3) | － |  | － |  |  |
| P-glycoprotein inhibitors | Yes | 421 | 3 (0.7) | 0.54 (0.17, 1.68) | 0.287 | 0.67 (0.22, 2.11) | 0.497 |  |
|  | No^*^ | 24,750 | 328 (1.3) | － |  | － |  |  |
| Dyslipidemia | Yes | 10,539 | 139 (1.3) | 0.99 (0.80, 1.24) | 0.953 | 0.99 (0.77, 1.27) | 0.921 |  |
|  | No^*^ | 14,632 | 192 (1.3) | － |  | － |  |  |
| Gastrointestinal disease | Yes | 7,061 | 85 (1.2) | 0.88 (0.69, 1.13) | 0.318 | 0.78 (0.59, 1.04) | 0.092 |  |
|  | No^*^ | 18,110 | 246 (1.4) | － |  | － |  |  |
| Antiplatelet agents | Yes (only one) agent) | 2,250 | 29 (1.3) | 0.99 (0.67, 1.45) | 0.946 | 0.92 (0.61, 1.41) | 0.718 |  |
|  | No^*^ | 22,281 | 294 (1.3) | － |  | － |  |  |
| Polypharmacy | <5 agents^*^ | 7,510 | 87 (1.2) | － |  | － |  |  |
|  | ≥5 agents | 16,934 | 236 (1.4) | 1.22 (0.96, 1.56) | 0.109 | 1.18 (0.88, 1.59) | 0.268 |  |
| Creatinine clearance | ≥15 mL/min to <30 mL/min  <30 mL/min | 618 | 11 (1.8) | 1.44 (0.79, 2.64) | 0.235 | 1.32 (0.68, 2.54) | 0.410 |  |
|  | ≥30 mL/min^*^ | 18,941 | 247 (1.3) | － |  | － |  |  |

^*^Reference.

^#^Sex, body weight, history of bleeding, type of AF, systolic blood pressure, severe liver dysfunction, diabetes mellitus, hyperuricemia, heart failure and/or reduced left ventricular ejection fraction, myocardial infarction, cerebrovascular disease, other thromboembolic disease, active cancer, dementia, fall within 1 year, history of catheter ablation, dyslipidemia, creatinine clearance, gastrointestinal diseases, polypharmacy (5 or more), anticoagulant, and use of antiarrhythmic agents, proton pump inhibitors, P-glycoprotein inhibitors, and antiplatelet agents were included as an adjustment factor in the model. Type of anticoagulants were included in the multivariate analysis model as an explanatory factor.

AF = atrial fibrillation; CI = confidence interval; DOAC = direct-acting oral anticoagulant; HbA1c = glycated hemoglobin; HR = hazard ratio; LVEF = left ventricular ejection fraction.
